# Flower heads in Asteraceae—recruitment of conserved developmental regulators to control the flower-like inflorescence architecture

**DOI:** 10.1038/s41438-018-0056-8

**Published:** 2018-07-01

**Authors:** Paula Elomaa, Yafei Zhao, Teng Zhang

**Affiliations:** Department of Agricultural Sciences, Viikki Plant Science Centre, University of Helsinki, P.O.Box 27, 00014 Helsinki, Finland

## Abstract

Inflorescences in the Asteraceae plant family, flower heads, or capitula, mimic single flowers but are highly compressed structures composed of multiple flowers. This transference of a flower-like appearance into an inflorescence level is considered as the key innovation for the rapid tribal radiation of Asteraceae. Recent molecular data indicate that Asteraceae flower heads resemble single flowers not only morphologically but also at molecular level. We summarize this data giving examples of how rewiring of conserved floral regulators have led to evolution of morphological innovations in Asteraceae. Functional diversification of the highly conserved flower meristem identity regulator LEAFY has shown a major role in the evolution of the capitulum architecture. Furthermore, gene duplication and subsequent sub- and neofunctionalization of *SEPALLATA*- and *CYCLOIDEA-*like genes in Asteraceae have been shown to contribute to meristem determinacy, as well as flower type differentiation—key traits that specify this large family. Future challenge is to integrate genomic, as well as evolutionary developmental studies in a wider selection of Asteraceae species to understand the detailed gene regulatory networks behind the elaborate inflorescence architecture, and to promote our understanding of how changes in regulatory mechanisms shape development.

## Introduction

Asteraceae, commonly referred as the sunflower or daisy family, has a prominent place in our daily life as it includes many economically important food crops (sunflower, lettuce, artichoke, endive, and safflower), herbal and medicinal species (*Calendula*, *Artemisia*, *Echinacea*), as well as popular ornamentals (gerbera, chrysanthemum, aster, dahlia, zinnia, and marigold). Also some noxious weeds, such as dandelion or thistle are members of the family. Asteraceae represents the largest family of flowering plants with around 25,000 species that are widespread into nearly all terrestrial habitats except of the Antarctica^1,2^. From evolutionary perspective, Asteraceae is a young family. Relatively recent discoveries of fossils have proposed scenarios that the split of Asteraceae from its ancestor occurred in Patagonia, southern South America some time before the Eocene 56–34 million years ago, and it spread to Africa before the two continents became geographically isolated^3,4^. The family then rapidly diversified and colonized the earth. This explosive tribal radiation has been associated with complex history of whole-genome duplication events, and especially with a paleopolyploidization event shared with Asteraceae and its sister family Calycearaceae, as well as a second round of genome duplications among the core Asteraceae tribes^5,6^. At the genetic level, functional analyses are necessary to explore how the ancestral polyploidization has contributed to the evolution of the extensive diversity of developmental traits, secondary metabolites, as well as life histories discovered in Asteraceae.

The key morphological innovation that has been associated with the evolutionary success of Asteraceae is the unique head-like inflorescence, capitulum that is the distinguishing feature of the whole family. Capitulum is a pseudanthium, a false flower that superficially mimics a single flower but is a highly aggregated structure comprised of multiple flowers with specialized functions. Asteraceae is largely pollinated by insects (rarely by birds), and several studies have indicated that the unique organization of the capitulum has selective advantage to pollinator attraction and plant vigor^7–9^. Moreover, the showy capitulum with all the diversity in shapes and colors generated by breeders is surely a key attraction to consumers contributing to the economic significance of this family in the ornamental industry. In this review, we aim to summarize the recent research in Asteraceae models to understand genetic regulation of capitulum development, as well as its organization and evolution. Altogether, the data demonstrate novel gene functions for both conserved floral regulators as well as duplicated genes that through sub- and neofunctionalization have gained specific roles in regulation of the elaborate inflorescence architecture.

## Organization of Asteraceae heads

**Fig. 1:**
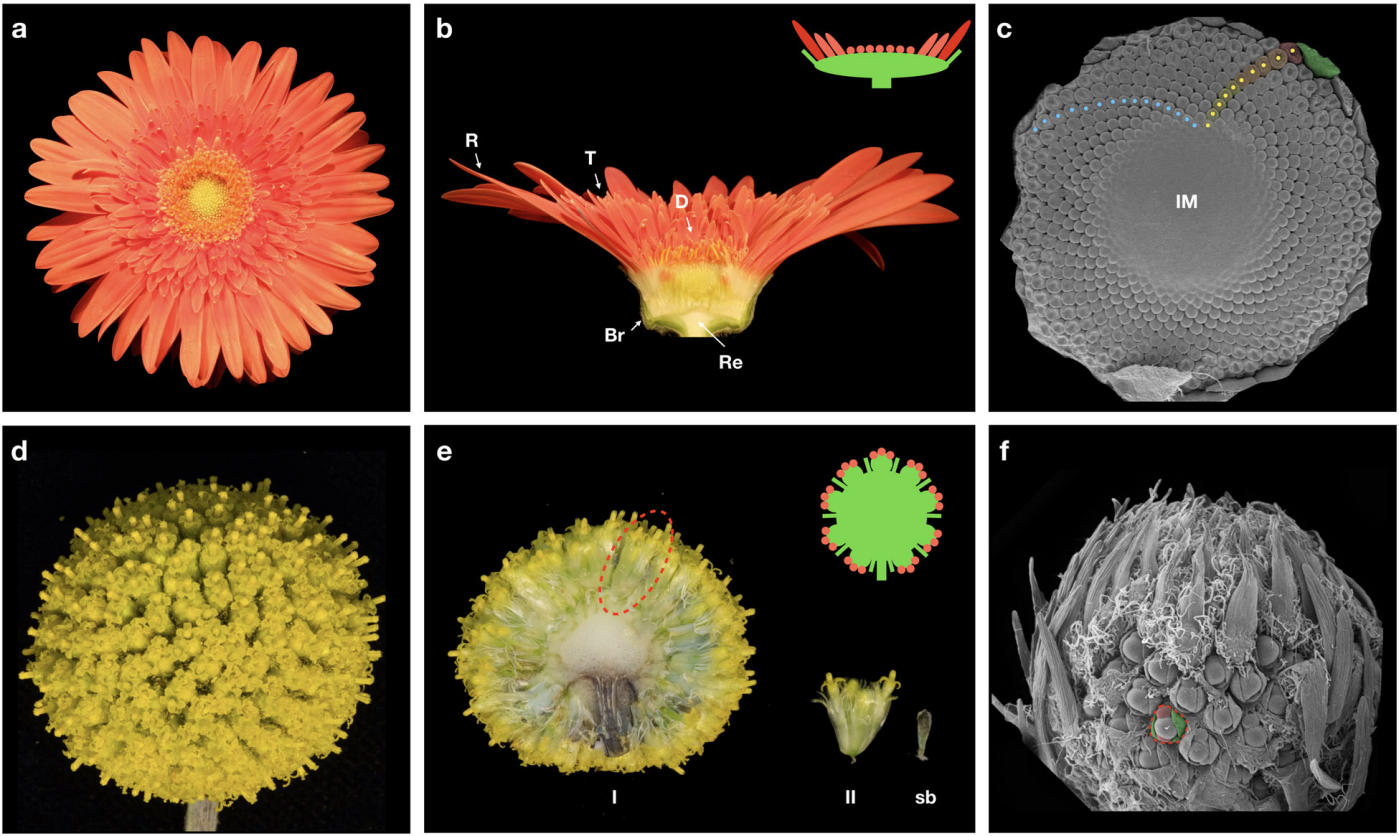
Organization of flower heads in Asteraceae. **a** Top view of a gerbera head. **b** Cross section of a gerbera head showing marginal ray (R), intermediate trans (T), and central disc (D) flowers that are all attached to a single, expanded receptacle (Re). The entire structure is surrounded by involucral bracts (Br) giving an impression of a single flower. **c** Scanning electron microscopy image focusing on early developmental stage of a growing head. The inflorescence meristem (IM) produces flower primordia in clockwise (blue dots) and counterclockwise (yellow dots) spirals. **d** Top view of a syncephalium of *Craspedia globosa*. **e** Cross section of a *Craspedia* primary head (I) shows multiple secondary heads (II, red circle) attached to a single receptacle, and developing a subtending bract (sb). **f** Scanning electron microscopy image of a *Craspedia* syncephalium at early developmental stage. A single-secondary head is highlighted (red circle) showing secondary inflorescence meristem producing bract (shaded in green) and flower primordia (shaded in red)

Asteraceae flower heads combine multiple flowers attached on a single receptacle (Fig. 1). After induction of flowering, the early ontogeny of the head is characterized by rapid expansion of the inflorescence meristem (receptacle), while later the meristematic area gradually shrinks and is fully consumed by emerging flower primordia^10,11^. In simple heterogamous, or radiate flower heads, the margin of the capitulum is occupied by showy ray flowers and the center with less conspicuous disc flowers. In sunflower (*Helianthus*), the marginal ray flowers are sterile and they develop extended ligules while the central disc flowers are perfect, and produce pollen. In gerbera (*Gerbera*) the ray flowers are female, and it may also develop intermediate trans flowers that resemble rays but are just smaller in size (Fig. 1a, b). In contrast, the homogamous heads are formed of only single-flower types. For example, the heads of lettuce (*Lactuca*) are composed of only ligulate ray flowers while the discoid heads in thistles, representing several genera in Asteraceae, develop solely disc flowers. The discoid capitula have been considered as an ancestral condition in Asteraceae^1,12^ where the emergence of ray flowers has occurred multiple times independently. The entire head is surrounded by involucral bracts that behave like sepals in single flowers, giving protection to the developing head at early developmental stages (Fig. 1b).

Asteraceae includes also species that show a higher level of complexity in their inflorescences. As examples of simple capitula, sunflower and gerbera heads appear as solitary structures on top of a floral stem. However, several capitula may also form synflorescences being arranged in distinct branched systems, such as racemes (e.g., *Artemisia pycnocephala*), cymes (e.g., *Senecio vulgaris*), or corymbs (e.g., *Achillea millefolium*)^13^. Another hierarchical level of complexity is generated when flower heads are aggregated onto a single receptacle, and form higher order structures, ‘heads within heads’, also known as capitulescence or syncephalium (e.g., *Craspedia*, *Echinops, Lagascea*)^14–16^ (Fig. 1d–f).

A characteristic feature shared by distinct head-like inflorescences is the spatial arrangement of individual flowers on the expanded receptacle (Fig. 1c). They appear in a phyllotactic pattern that follow strict clockwise and counterclockwise spirals (contact parastichies), the numbers of which always follow the two consecutive numbers in the mathematical Fibonacci series: 1, 1, 2, 3, 5, 8, 13, 21, 34, etc. where each number is a sum of the two previous ones. The spirals can usually reach high numbers; for example flowers on gerbera and sunflower heads are typically arranged in 34/55 and 55/89 spirals, respectively. The initiation of individual flowers in a capitulum has been thought to occur in an acropetal manner, from the margins toward the center of the capitulum. This is indeed the case in homogamous heads with only single-flower types, however, morphological studies have indicated that in heterogamous heads the early ontogeny of marginal ray flower primordia is distinct^10,13^. In fact, in extreme cases, ray flower development may proceed basipetally toward the margins of the head (instead of the center), or in milder cases ray primordia show a developmental arrest compared to the development of an adjacent disc or trans flower primordia^10,13,17–19^. This differential early ontogeny has turned out to be crucial for understanding how distinct flower types have evolved (see below).

## Flower meristem identity gene functions give light for understanding the evolution of the capitulum architecture

Inflorescences are typically branched structures that bear flowers. As known from the conventional model species, the major difference between the two basic types of inflorescences, racemes in *Arabidopsis* or *Antirrhinum*, and cymes in Solanaceae species such as petunia or tomato, is in their determinacy^20^. The elongating, indeterminate racemes continuously produce flowers in the flanks of the inflorescence meristem (IM) while in cymes the meristem always terminates in a flower, but forms a new axillary IM that continues the growth. Two highly conserved genes *LEAFY* (*LFY*) and *UNUSUAL FLORAL ORGANS* (*UFO*), encode the key regulators that define flower meristem (FM) identity. However, these genes function in an opposite manner in racemes and cymes^20,21^. In Arabidopsis, *LFY* expression is specifically localizing to FMs^22^, and through interaction with UFO^23^ and SEPALLATA3 (SEP3)^24^, LFY activates flower organ identity genes, and floral developmental program. In cymes, instead, the FM identity is defined by the *UFO* homologs, *DOUBLE TOP* (*DOT*) in petunia^25^ or *ANANTHA* (*AN*) in tomato^26^.

The unique, and apparently successful, organization of the capitulum has inspired botanists to investigate its evolutionary origin. Capitulum is often interpreted as a compressed raceme or cyme^27^. However, Claβen-Bockhoff and Bull-Hereñu^28^ proposed that it has evolved from a single, determinate meristem that through subdivision gave rise to multi-flowered head. Claβen-Bockhoff and Bull-Hereñu present of concept of a floral unit meristem (FUM) that resembles a single flower meristem (FM) by its histological organization and development, being flat and enlarged structure with inhibited internodes. The distinction of FUM is that it produces flower primordia in an acropetal manner while a FM develops floral organ primordia. An alternative scenario was presented based on morphological studies on the basal relatives of Asteraceae (Menyanthaceae, Goodeniaceae, and Calyceraceae) where the main axis of the inflorescence shows a racemous, and the basal lateral branches cymous branching pattern^29,30^. According to Pozner et al.^30^ Asteraceae heads evolved from these mixed inflorescences through the loss of the marginal, cymose branches (called cymose units) and a terminal flower still found in the compressed inflorescences (cephalioids) of Calyceraceae, a sister family to Asteraceae. The marginal ray flowers are thus considered to originate from the cymose units, experiencing further functional differentiation.

**Fig. 2:**
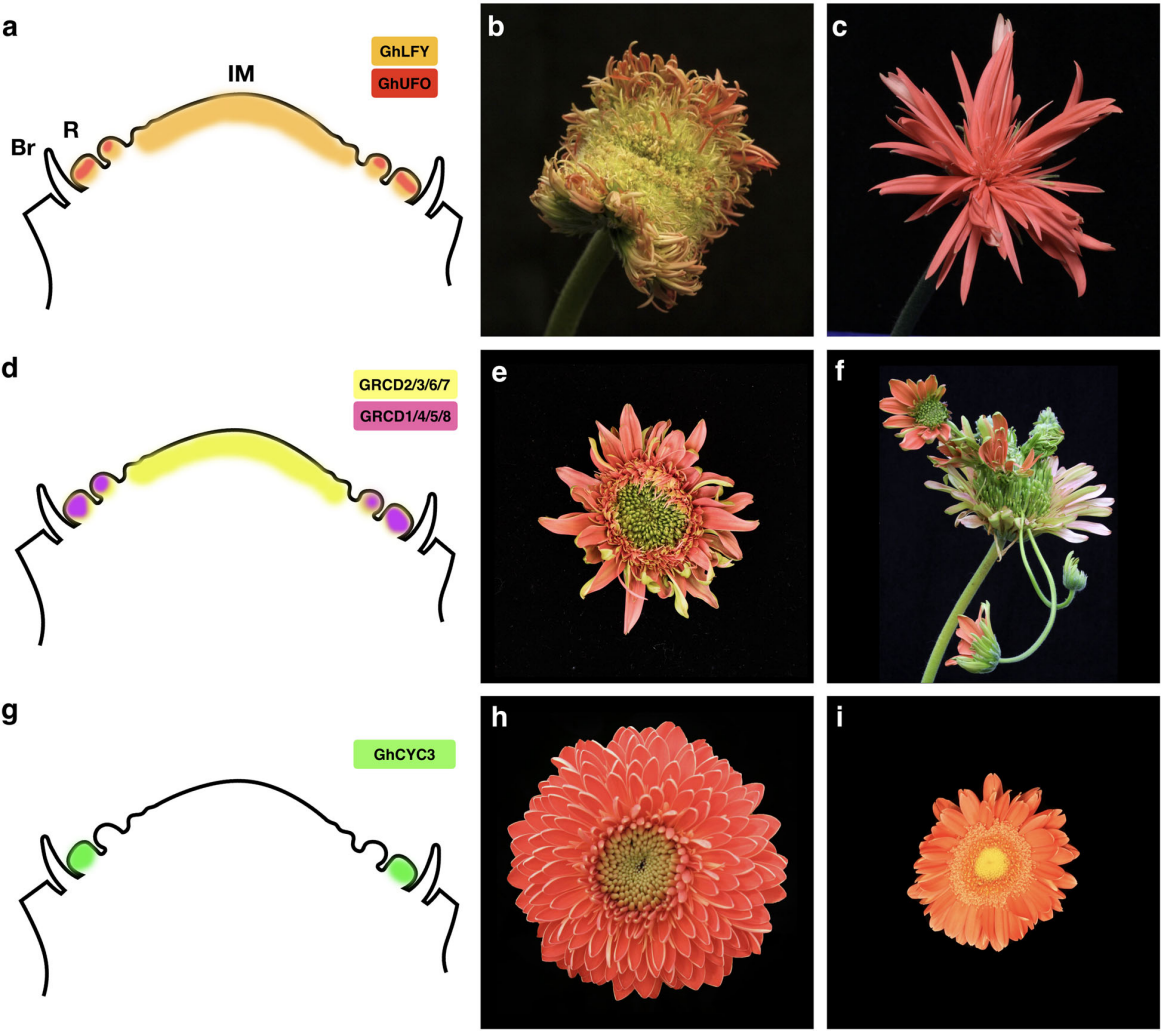
Schematic representation of expression domains, and examples of representative transgenic phenotypes for key regulatory genes affecting head development in gerbera. **a** The expression of flower meristem identity gene *GhLFY* is detected both in undifferentiated inflorescence meristem (IM) as well as in emerging flower primordia while *GhUFO* expression in localizing to flower primordia^10^. **b** Transgenic phenotype of a *GhLFY* RNAi line showing loss of organ identity. **c** Ectopic expression of *GhUFO* converts the meristem into a single flower that develops only multiple flower organs instead of florets. **d** The expression domains of the duplicated *SEP*-like *GRCD* genes. **e** A mild phenotype of a transgenic *GRCD4/5* double RNAi line shows defects in petal development. **f** Downregulation of multiple *GRCD* genes lead to floral reversion where carpels or ovaries of individual flowers are replaced by developing new heads. **g** *CYC2* clade gene *GhCYC3* is specifically expressed in ray flower primordia. **h** A crested gerbera cultivar develops only ray flowers as a result of ectopic expression of *GhCYC3* throughout the capitulum. **i** Transgenic gerbera *GhCYC5* RNAi lines shows significantly reduced petal length in ray and trans flowers

In order to test these distinct hypotheses of the evolutionary origin of flower heads in Asteraceae, Zhao et al.^10^ conducted functional studies of FM gene orthologs in gerbera. As an assumption, loss of FM identity would occur similarly in both flower types if they originated from a single meristem, or differentially, if they originated form distinct branching system. Most interestingly, and in contrast to conventional model species, the gerbera *GhLFY* showed uniform expression across the entire, naked IM (Fig. 2a). This pattern defines the capitulum as a determinate structure with resemblance of single FM. Suppression of *GhLFY* expression in transgenic RNAi lines led to loss of flower organ identity, as expected (Fig. 2b). However, the lines also revealed a specific role for *GhLFY* in regulation of ray flower development. The ray primordia were converted into branched units resembling the marginal cymose units as found in Calyceraceae. Moreover, *GhLFY* expression domain was found to be associated with the early ontogeny (developmental delay) of ray flowers emerging at the axils of involucral bracts. The gerbera *GhUFO* instead showed FM-specific expression, and a conserved role in regulating the FM identity (Fig. 2a). Ectopic expression of *GhUFO* was sufficient to convert the capitulum into a single flower associated with a dramatic change from spiral to whorled phyllotaxis, and development of numerous organ primordia instead of flowers^10^ (Fig. 2c).

Altogether, the molecular studies in gerbera suggest that none of the previous hypotheses of patterning and evolution of the capitulum architecture are sufficient by themselves. In fact, the current data gives support for both hypotheses. The differential role *GhLFY* in controlling flower type identity suggests that floral dimorphism is connected with their distinct origin from separate branching systems^10,30^. However, it cannot be excluded that the centre of the capitulum, harboring the disc flowers, may indeed have originated by subdivision of a single meristem as proposed by Claβen-Bockhoff and Bull-Hereñu^28^. As one possibility, this could have occurred as a result of temporal changes in *GhUFO* expression needed to define FM identity. Most importantly, the data also emphasizes that the capitulum can be seen as an analog of a single flower not only morphologically but also at molecular level. Comparative evo-devo studies should be extended to the basal relatives of Asteraceae as well as to syncephalious species to clarify the current hypotheses, and to better understand the molecular networks behind this vast morphological diversity.

## Gerbera *SEPALLATA*-like gene functions in regulation of IM determinacy

Based on the absence of a clearly defined, single terminal flower, earlier botanical studies have classified flower heads as indeterminate inflorescences^13,31^. However, functional studies of the gerbera *GhLFY* indicate that the expanding IM is in fact determinate, and eventually fully packed with flower primordia^10,32^. In transgenic lines with suppressed *GhLFY* expression, the IM never got fully consumed with emerging primordia that were randomly initiating in the center of the capitulum^10^. Furthermore, functional studies have indicated that also the E class *SEPALLATA*-like MADS box genes, *GERBERA REGULATOR of CAPITULUM DEVELOPMENT* 2/7 (*GRCD2/7*), are required for the determinacy of the IM^11,33^. The number of the flowers produced in the inflorescences of gerbera antisense-*GRCD2* lines were nearly doubled compared to the wild type, and this was attributed to the indefinite growth of IM that remained undifferentiated until senescence^33^. Similar phenotypes were observed in transgenic gerbera lines when *GRCD7*, a close paralog of *GRCD2*, was downregulated^11^. Both *GRCD2* and *GRCD7* belong to the SEP1/2/4 clade (also known as LOFSEP clade) of E function genes^11,34,35^. Like *GhLFY*, both *GRCD2* and *GRCD7* show ubiquitous expression in the undifferentiated IM throughout the early ontogeny of flower heads^11^ (Fig. 2d).

*LFY* gene is present as a single copy gene in most of the land plant species^21^. It is an example of a gene in Asteraceae that has evolved a novel function to regulate early ontogeny of ray flowers as well as determinacy of the capitulum^10^ (Fig. 2a). In contrast, the *SEP*-like *GRCD* genes are represented by a medium size gene family, composed of altogether eight members in gerbera^11,35^. In contrast to high level of redundancy among the four *Arabidopsis SEP* genes, affecting the identity of all organ whorls^36,37^, the gerbera *GRCD* genes show subfunctionalization. At the level of individual flowers, *GRCD* genes have evolved whorl-specific functions, with distinct gene family members contributing to the identity of stamens, carpels as well as petals, respectively^11,33,38,39^ (Fig. 2d, e). Furthermore, similarly as *SEP* orthologs in *Arabidopsis*, petunia or tomato^37,40,41^, *GRCD* genes show conserved roles in regulating the maintenance of flower meristem identity. In extreme cases, loss of *GRCD* expression led to floral reversion replacing carpels or ovaries by a new capitulum^11^ (Fig. 2f). What is specific to Asteraceae is that the duplicated *SEP*-like genes have been recruited to regulate IM development, an example of potential neofunctionalization. In addition to *GRCD2* and *GRCD7*, two other genes, *GRCD6* of the *SEP1/2/4* clade, and *GRCD3* of the closely related *AGL6* clade were identified as candidates to regulate IM patterning in gerbera^11^ (Fig. 2d). However, their detailed functions are not yet defined. The determinate fate of IM in flower heads could be, at least partially, explained by the temporal and spatial changes in expression and functions of these floral meristem/organ identity genes contributing to their novel roles in regulating Asteraceae inflorescence architecture^10,11^.

Understanding of patterning of the Asteraceae IM still awaits answers for many open questions. How is the determinacy of the IM established? What are the genetic factors up- and downstream of GRCD2/7 and GhLFY? A determinate meristem is programmed to terminate its activity in a precise manner, at correct time and location. This is exemplified in the *Arabidopsis* flower meristem, where termination is executed through the activity of AGAMOUS (AG) in its central domain leading to suppression *WUSCHEL* (*WUS*) expression, a key gene to promote stem cell activity^42–44^. Aerial meristems in *Arabidopsis* are histologically following a zonal arrangement with separate central, peripheral and rib zones^45^. Stem cells of the meristem are maintained within the central zone, through a well-known signaling cascade involving a negative feedback loop between WUS and CLAVATA3 (CLV3) peptide^46–48^. In contrast, the IM in Asteraceae flower heads is organized in a distinct’mantle-core’ configuration, consisting an enlarged meristematic mantle in addition to a quiescent core underneath^45,49^. As necessary factors for maintaining the determinate fate of the IM in heads, GRCD2/7 and GhLFY must have master roles in regulating the genetic program that determines first the shrinkage of the meristematic volume, and later its consumption and termination. So far, we still lack the genetic evidence to explain how stem cell homeostasis is regulated in the mantle-core configurated meristem.

## *CYCLOIDEA2* clade genes define the identity of the marginal ray flowers

The presence of distinct flower types is a characteristic feature of Asteraceae, and so far mostly studied trait at genetic level. Typically, the flower types differ in their symmetry—marginal ray (and trans) flowers being bilaterally symmetrical (zygomorphic) and central disc flowers radially symmetrical (actinomorphic) (Fig. 1a, b). At the level of single flowers, the symmetry regulation by CYCLOIDEA/TEOSINTE BRANCHED1-like TCP domain transcription factors is well-established across core eudicots^50,51^. During the last decade, dissection of functions of the *CYC/TB1*-like genes in *Gerbera*, *Helianthus, Senecio*, and *Chrysanthemum* indicate their independent recruitment in defining flower type identity in Asteraceae^52–59^. This function can specifically be addressed to the CYC2 subclade gene family members that have experienced frequent duplications in Asteraceae^53,54,56,59^. The CYC2 clade homologues are predominantly expressed in the periphery of the capitulum, in emerging ray flower primordia while low, or no expression is detected in the central disc primordia^17,53,55,56,58,59^ (Fig. 2g). The functional data indicate that ray flower identity is controlled by different *CYC2* paralogs in distinct Asteraceae lineages^54,56,58^ giving molecular support that ray identity evolved multiple times independently in the family^60^. Interestingly, duplicated *CYC/TB1*-like genes have also been identified in Dipsacaceae^61^ and Myrtaceae^62^ where pseudanthium-type inflorescences with distinct flower types originated independently of Asteraceae.

Functional studies have shown variability in phenotypes of transgenic plants and/or in mutant lines indicating that, although *CYC2* homologues clearly affect flower type differentiation, they may function in a species-specific manner (Table 1). Conversion of disc flowers into ray-like, with elongated ligules and disrupted stamen development, was discovered in transgenic gerbera by ectopic activation of GhCYC2, GhCYC3 and GhCYC4, respectively^52,57^. In the so-called crested or double-flowered cultivars of gerbera (Fig. 2h) and *Chrysanthemum morifolium*, as well as in *double-flowered* (*dbl*) and *chrysanthemoides* (*Chry*) mutants of sunflower, all flowers are of ray identity. All these phenotypes have been shown to arise by ectopic activation of CYC2 clade genes throughout the capitulum^55,57,59,63^. Interestingly, in *Senecio* and chrysanthemum, ectopic expression of ray-specific CYC genes did not affect disc flower development^53,59^. Furthermore, also the ligule length of ray and/or trans flowers is differentially affected by genetic transformation in distinct species^52,53,59^ (Table 1) indicating that the given genes may function in distinct regulatory networks. So far, gene silencing has been challenging, and has not led to complete absence of ray flowers suggesting genetic redundancy among the gene family members, or involvement of yet unknown factors (Fig. 2i). The development of trumpet shaped, actinomorphic ray flowers has been associated with reduced *CYC* gene expression in *tubular-rayed* (*tub*) sunflower mutant^55,64^, and in genus *Anacyclus* (tribe Anthemidae)^17^ but also with ectopic expression as the *Senecio RAY2*^53^. Further functional studies, e.g. with CRISPR/Cas9 approaches, are necessary to study whether complete suppression of CYC2 activity is sufficient to fully abolish ray flower initiation or whether it only affects organ differentiation.

**Table 1:** Summary of phenotypes in transgenic lines, cultivars and mutants in Asteraceae family affected by *CYC2* clade *TCP* genes

Species	Modification/mutant		Observed phenotype		
			Ray	Trans	Disc
*Gerbera hybrida*	Ectopic expression of *GhCYC2, GhCYC3 or GhCYC4*^52,57^		Reduced ligule length	No effect	Ray-like with elongated ligules and disrupted stamen development
	Cosuppression of *GhCYC2*^52^		No effect	Reduced ligule length, occasional splitting of ligules	No effect
	Crested cultivar CH02.663: upregulation of *GhCYC3* expression in disc flowers^57^		Ray flower identity only		
*Senecio vulgaris*	Ectopic expression of *RAY1*^53^		Reduced ligule length, complete absence of ray flowers	—	No effect
	Ectopic expression of *RAY2*^53^		Tubular ray flowers by promoting elongation of all petal lobes	—	No effect
	Suppressed expression of *RAY3*^58^		Reduced ligule length	—	No effect
*Chrysanthemum lavandulifolium*	Ectopic expression of *CmCYC2c*^59^		Increased ligule length	Decreased ligule length	No effect
*Chrysanthemum morifolium*	‘Double-ray flowered’ heads: upregulation of *CmCYC2c* expression in disc flowers^59^		Ray flower identity only		
*Anacyclus clavatus*	Trumpet-type: low expression of *AcCYC2b*, *AcCYC2c* and *AcCYC2d* in the ray flowers^17^		Tubular ray flowers	—	No effect
*Helianthus annuus*	*Double-flowered* (*dbl*)	Ectopic expression of *HaCYC2c* by an insertion in the promoter region of *HaCYC2c*^55,63,79^	No effect	—	Ray-like
	*Chrysanthemoides* (*Chry*)		No effect	—	Ray-like, reduction in stamen length, absence of ovules
	*Tubular-rayed* (*tub*)	Inactivation *HaCYC2c* by an insertion of transposable element in the coding region of *HaCYC2c*^6,55,64^	Tubular ray flowers	—	No effect
	*Tubular ray flower* (*turf*)		Tubular ray flowers, fertile stamens and ovules	—	No effect

The role of CYC-like proteins in regulating flower symmetry was originally discovered in bilaterally symmetrical flowers of *Antirrhinum majus*^65,66^, as also their interaction with MYB-domain regulators RADIALIS (RAD) and DIVARICATA (DIV)^67^. In *Antirrhinum*, CYC defines the identity of the dorsal domain of the flowers by promoting the enlargement of the dorsal petals and disrupting the development of the dorsal stamen^65,66^. CYC is positively regulating *RAD*^68^ whereas DIV, that defines the identity of ventral petals, is repressed by RAD from the dorsal domain^67^. Recent studies in *Senecio* suggest that similar regulatory network exists in Asteraceae but involves diversified expression domains of these key regulatory genes as well as modifications in their interactions^58^. Functional studies indicate that the *Senecio RAY3* promotes elongation of the large ventral ligule of ray flowers while *SvDIV1B* apparently functions as a negative regulator of elongation by inhibiting *RAY3* and *SvRAD*. Garcês et al.^58^ further suggest complex regulatory relationships between the *CYC2* genes, and identified two other *CYC2* clade genes, *RAY1* and *RAY2*, as potential target genes of RAY3. However, detailed understanding of the network still awaits further functional studies.

The pairwise protein–protein interaction studies conducted with gerbera and sunflower indicated that CYC/TB1-like proteins function in complexes that are likely to direct their functional specificity^56^. Interestingly, the capacity of CYC/TB1-like proteins to form homodimers varied between gerbera and sunflower while heterodimer formation was more similar between the two species that represent distant tribes in Asteraceae. Positive autoregulatory feedback loops of CYC2 clade genes themselves as well as cross-regulation among each other has been identified in Gesneriaceae (Lamiales) as a mechanism for maintaining *CYC2* gene expression in the dorsal domain of the developing flowers^69^. Cross-regulation has already been suggested for the *RAY* genes in *Senecio*^58^ as well as for *CmCYC2* genes in *C. morifolium*^59^. Further studies are still required to identify potential upstream as well as interacting regulators of CYC2 clade genes. The mechanisms of how CYC2 clade genes acquire their specificity to regulate early flower type determination as well as later stages of organ differentiation are fully unknown. Transcriptome analyses in gerbera and chrysanthemum have revealed candidate transcription factor families, including MADS-box proteins, but also members of NAC, MYB, AP2/ERF, WUS, CYC2, and other TCP families, among differentially expressed genes between the ray and disc flowers^70,71^, however, their connections with CYC2 proteins have not been established.

## Future prospects

The advances in next-generation sequencing already have, and still will dramatically change biological research. For Asteraceae, the reference genome sequences for lettuce (*Lactuca sativa*)^72^ and sunflower^73^, as well as draft sequences for horseweed (*Conyza canadensis*)^74^, globe artichoke (*Cynara cardunculus*)^75^, and sweet wormwood (*Artemisia annua*)^76^ have been published. Genome information has established the evolutionary history of the family. Sunflower, lettuce, and artichoke have experienced a whole-genome triplication (WGT-1, 38-50 Mya) event in addition to the ancestral WGT-γ (gamma triplication, 122–164 Mya) common for eudicots^72,73^. Moreover, younger, lineage specific duplications (WGD-2, 29 Mya) have occurred in sunflower^73^, and also in the gerbera lineage^5^. As indicated for gene regulatory networks of flowering time in sunflower^73^, these polyploidization events have resulted in more complex regulatory networks, involving additional paralogs in Asteraceae compared to the *Arabidopsis* model. Also in lettuce, enrichment of some transcription factors and DNA-binding proteins in the triplicated regions of the genome, as well as sequence divergence was discovered^72^. This is also apparent for the specific developmental regulators reviewed here, highlighting the value of Asteraceae as a model to understand evolution of gene functions through gene duplication and subsequent diversification.

Detailed understanding of floral and inflorescence diversity both at evolutionary and developmental context is of utmost importance as they form the basis for plant reproduction, yield and human sustenance. Evolutionary developmental (evo-devo) approaches combine plant systematics (phylogenetics), developmental genetics and genomics in an unprecedented way^76^. In a context of a robust phylogenetic framework for angiosperms, large sequence datasets are produced not only from single-model species, but from several species that represent the phenotypic variation across ‘model clades’^77^. Asteraceae clearly represents yet not very extensive studied, but promising and emerging model clade in this respect. Recently, genome skimming, or whole-genome shotgun sequencing, as a low-coverage and cost-effective method was used for mining of low-abundant *CYC*-like genes from 24 Goodeniaceae species, basal relatives of Asteraceae^78^. The possibility for simultaneous comparisons of multiple taxa in association of trait gains, losses, or modifications will facilitate our understanding of how changes in regulatory mechanisms shape development^78^. Combined with functional studies, for example in well-established models, this will also pave the way for new applications in breeding of novel traits in important agricultural and ornamental crops.
